# Complete Genome Sequence of Citrobacter rodentium Strain DBS100

**DOI:** 10.1128/MRA.00421-19

**Published:** 2019-06-13

**Authors:** Georgy Popov, Aline Fiebig-Comyn, Steven Shideler, Brian K. Coombes, Alexei Savchenko

**Affiliations:** a Department of Microbiology, Immunology and Infectious Diseases, University of Calgary, Calgary, Alberta, Canada; b Department of Biochemistry and Biomedical Sciences, McMaster University, Hamilton, Ontario, Canada; Broad Institute

## Abstract

Citrobacter rodentium strain DBS100 causes an infection of the intestines in mice. It provides an important model for human gastrointestinal pathogens, such as enteropathogenic and enterohemorrhagic Escherichia coli, which cause life-threatening infections. To identify the genetic determinants that are common across the enteropathogenic bacteria, we sequenced the DBS100 genome.

## ANNOUNCEMENT

Citrobacter rodentium strain DBS100 is a Gram-negative murine enteropathogen (a generous gift from Brett Finlay, University of British Columbia) which is closely related to clinically important enteropathogenic and enterohemorrhagic Escherichia coli strains (1). The infection of mice by DBS100 is used as a model to elucidate the virulence mechanisms employed by bacterial enteropathogens to colonize the intestinal tract of their host (1). Mice orally infected with DBS100 develop colonic hyperplasia, infiltration of immune cells into the infection site, depletion of goblet cells, diarrhea, and lesions on the intestinal epithelia (1). DBS100 induces the formation of pedestal-like structures on the epithelial cell surface which support the pathogen’s noninvasive attachment to the host (1). To identify genetic determinants associated with entropathogenicity, we sequenced the DBS100 genome.

DBS100 was grown in Luria broth liquid medium at 37°C, and total genomic DNA (gDNA) was extracted using the DNeasy Ultraclean microbial kit (Qiagen, USA). The gDNA was sheared using Covaris sonication and processed with the SMRTbell template prep kit v1.0 (Pacific Biosciences, Canada) using the manufacturer’s protocol, and 58,398,237 bp were sequenced with Pacific Biosciences RS II sequencing technology using one single-molecule real-time (SMRT) cell. The raw PacBio reads (genome coverage, 267×) were processed using the Hierarchical Genome Assembly Process (HGAP) workflow (2) with a cutoff of 30× to generate 6,848 corrected long subreads with minimal, average, and maximal read lengths of 500, 8,527, and 27,770 bases, respectively. The corrected long subreads were assembled into four contigs of 31,068, 42,783, 73,049, and 5,343,648 bp (genome coverage, 10.6×) using SMRT Analysis v2.3.0.140936.p5 (Pacific Biosciences). To close the circular chromosome of DBS100, we used PCR amplification of the 3,177-bp fragment between the 5′ and 3′ ends of the 5,343,648-bp contig using the Quick-Load Taq 2× master mix (New England BioLabs, Canada) and primers CCCTTTGAACCCAGGCTACG and CGTCAACATCCGGGTTATAGCG, and we sequenced this fragment using Sanger sequencing technology. Next, DBS100 gDNA was extracted using phenol chloroform treatment following purification and concentrated via ethanol precipitation (3). RNA contaminants were removed using RNase Cocktail (Invitrogen, USA) following the manufacturer’s protocol. The gDNA was fragmented using Covaris sonication and processed with the NEBNext Ultra II library preparation for Illumina kit (New England Biolabs, USA) using the manufacturer’s protocol, and 352.97 Mbp were sequenced with Illumina MiSeq v2 technology (2 × 150-bp paired ends). The adapter sequences at the 5′ ends and 10 bases at the 3′ ends of the raw Illumina reads were trimmed using Cutadapt v1.15 (4) to obtain 1,176,564 short reads (genome coverage, 27×) with minimal, average, and maximal lengths of 40, 127.28, and 136 bases, respectively. The trimmed short reads were analyzed for sequence quality using FastQC v0.11.5 (quality control score > 20) (5). Hybrid assembly of the Illumina-derived short reads and PacBio-derived contigs was done using Unicycler v0.4.7 (6), SPAdes v3.12.0 (7), Minimap (8), and Pilon v1.23 (9) to generate nine contigs (*N*_50_, 5,232,658 bp). The following four out of the nine contigs were identical to previously reported plasmids: pCROD1 (GenBank accession number FN543503), pCROD2 (FN543504), pCROD3 (FN543505), and pCRP3 (NC_003114). The linear contigs corresponded to the large circular contig derived from the PacBio sequencing and were used to finalize the sequence of the strain DBS100 chromosome. The NCBI Prokaryotic Genome Annotation Pipeline (best-placed reference protein, v4.8) was used to annotate 5,186 genes in the 5,346,827-bp-long contig with a 54.69% GC content DBS100 chromosome. Out of the 5,186 genes, 4,788 are protein coding genes, 116 are RNA coding genes, and 282 are pseudogenes.

### Data availability.

The chromosomal genome sequences have been deposited in GenBank under the accession number CP038008. The sequences of the PacBio-filtered reads and Illumina raw reads have been deposited in the NCBI Sequence Read Archive (SRA) under the accession number PRJNA527323.
